# Improving Access to Child and Adolescent Weight Loss Surgery: A Review of Updated National and International Practice Guidelines

**DOI:** 10.7759/cureus.38117

**Published:** 2023-04-25

**Authors:** David Elkhoury, Christina Elkhoury, Vasavi Rakesh Gorantla

**Affiliations:** 1 Anatomical Sciences, St. George's University, St. George's, GRD; 2 Molecular Pharmacology and Toxicology, University of Southern California, Los Angeles, USA

**Keywords:** body mass index (bmi), gastric bypass surgery, child nutrition, adolescent obesity, bariatric surgery

## Abstract

The rise of childhood obesity is a growing concern due to its negative impact on health. Metabolic bariatric surgery (MBS) has gained popularity as an effective and adequate intervention for children and adolescent patients living with severe obesity. Nonetheless, access to MBS for this population is still limited. The objective of this paper is to conduct a comprehensive review of the latest national and international practice guidelines and improve access to MBS for children and adolescents. The paper focuses on the recommendations from the 2023 American Academy of Pediatrics (AAP) and 2022 guidelines from the American Society for Metabolic and Bariatric Surgery (ASMBS) and the International Federation for the Surgery of Obesity and Metabolic Disorders (IFSO). Recently updated guidelines from the ASMBS and IFSO aim to improve access to MBS for children and adolescents and recommend patient selection, preoperative evaluation, and postoperative care. While lifestyle changes, medication, and behavioral therapy are commonly prescribed, they often fail to achieve permanent weight loss and its maintenance. Weight-loss surgeries like sleeve gastrectomy (SG) and gastric bypass (RYGB) show promising results in managing severe obesity in adolescents. SG has become the preferred method for treating severe obesity in adolescents, surpassing RYGB. Weight stigma is also explored in this review, revealing its negative effects on individuals who are overweight and underweight. Furthermore, telehealth is identified as an increasingly valuable tool for managing pediatric obesity, as it can improve access to care, particularly for those in remote areas where physicians trained to treat childhood obesity and the shortage of bariatric surgeons experienced in treating younger adolescents and pediatricians with advanced training are major obstacles.

## Introduction and background

Obesity among children is a growing interest in the United States and worldwide, with approximately one out of five children considered overweight and over 340,000,000 children and adolescents considered obese, according to a World Health Organization (WHO) report in 2016 [1,2]. Additionally, 8.5% of American adolescents, or 4.5 million individuals aged 12 to 19 years, are classified as morbidly obese with a body mass index (BMI) ≥120, 95th percentile [1]. Severe childhood obesity is linked to immediate health complications such as type 2 diabetes and adolescent hypertension, as well as potential downstream adult diseases such as sleep apnea and non-alcoholic steatohepatitis [3]. While lifestyle changes, including exercise and diet plans, are the foremost treatment for obesity, they may not be sufficient for some children and adolescents. In these cases, metabolic bariatric surgery (MBS) is considered a viable solution to the epidemic of childhood and adolescent obesity.

The WHO defines adolescence as the developmental stage between childhood and adulthood, typically covering the ages of 10 to 19 years. Several high-quality prospective studies have been conducted on adolescents with MBS [4]. Class II or greater obesity has been found to have significant impacts on prospective health. These consequences can include increased mortality risk and greater susceptibility to various illnesses. However, surgical programs present to be a successful and effective intervention for young individuals with severe obesity, irrespective of the presence of weight-related comorbidities. Such programs have been found to yield improvements in health, disease prevention, and prolonged life expectancy [5].

Despite the benefits of MBS, the pursuit of surgery has been limited due to challenges in access. To address this issue, the American Academy of Pediatrics (AAP), American Society for Metabolic and Bariatric Surgery (ASMBS), and the International Federation for the Surgery of Obesity and Metabolic Disorders (IFSO) have collectively released updated guidelines [6,7]. These guidelines aim to improve access to MBS for children and adolescents and provide recommendations on patient selection, preoperative evaluation, and postoperative care.

## Review

National and international practice guidelines

The 2023 AAP guidelines recommend that children and adolescents who have severe obesity defined as a BMI of 40 or higher, or a BMI of 35 or higher with significant health complications related to obesity, undergo weight loss surgery. In addition, the guidelines recommend that surgery should be considered after a comprehensive evaluation by a multidisciplinary team and implementation of a referral program designed to direct patients to facilities that provide specialized focused metabolic and bariatric surgical services [6]. Despite robust evidence indicating the safety and efficacy of MBS, it remains underutilized among the pediatric population [8]. Young people who are thinking about having metabolic or bariatric surgery are held to stricter criteria than adults. Adolescents with a BMI of 40 kg/m^2^, or a BMI of 35 kg/m^2^ who do not have or have only mild comorbidities, may be candidates for bariatric surgery [9].

**Figure 1:**
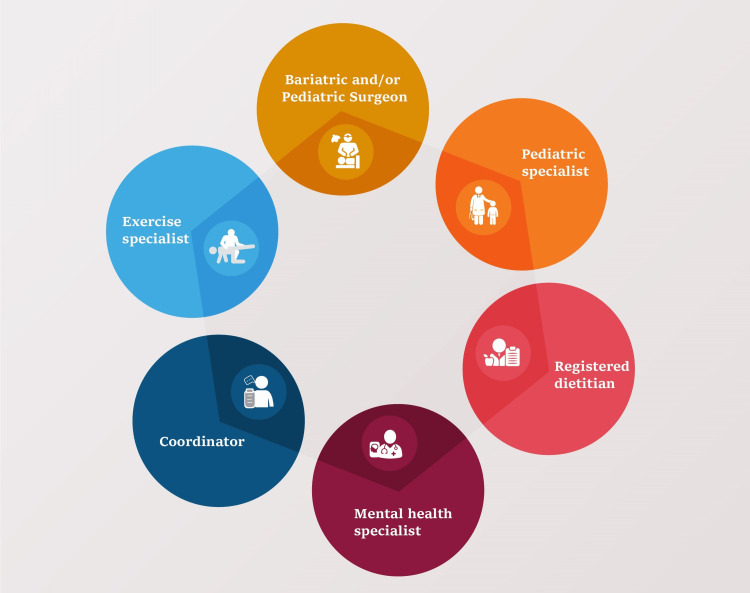
A Multidisciplinary Approach to Bariatric Surgery in the Pediatric and Adolescent Population. Adapted from the American Society for Metabolic and Bariatric Surgery (ASMBS) Public Education Committee and the ASMBS Pediatric Committee [10].

The 2022 ASMBS and IFSO guidelines also recommend children and adolescents with severe obesity defined as a BMI of 40 or higher, or a BMI of 35 or higher with significant obesity-related health complications, undergo weight loss surgery. The guidelines recommend that the surgery be conducted by a surgeon trained specifically in pediatric bariatric surgery [7]. The ASMBS also recommends a multidisciplinary approach, including the preoperative evaluation and postoperative follow-up by a team of healthcare professionals, as demonstrated in Figure 1.

Discussion of MBS for use in children and adolescents

During the past couple of decades, guidelines for the diagnosis of MBS in children have been developed, with a focus on eligibility and preoperative criteria. Historically, surgical intervention has only been considered after exhausting all other weight loss strategies [11]. In 2022, the IFSO and ASMBS issued updated guidelines, which focused on lower BMI requirements and replaced the 30-year-old guidelines issued by the National Institutes of Health in 1991 [9]. Recent clinical practice standards from the European Association for Endoscopic Surgery are very similar to the recommendations put forward by the ASMBS. Possible candidates for MBS include individuals with a BMI of 40 kg/m^2^, those with a BMI of 35-40 kg/m^2^ and comorbidities, and those with a BMI of 30-35 kg/m^2^ who have poorly managed type 2 diabetes possibly with arterial hypertension despite adequate pharmacological therapy [8].

Obesity in childhood and adolescent development is a serious public health concern as it often persists into adulthood, leading to a host of health problems. Despite various treatments, including lifestyle changes and pharmacological therapies, significant weight loss and maintenance have proven to be challenging [12]. Harmful effects on the physical and mental health of individuals as a result of obesity have been well documented, including increased mortality rates and a higher risk of developing serious mental health issues [13]. Moreover, research has shown that treating obesity in childhood and adolescence is particularly important to prevent the constancy of obesity into adulthood and to mitigate associated health risks [14]. Therefore, effective interventions for obesity during childhood and adolescent development are critical for preventing long-term health consequences.

**Figure 2:**
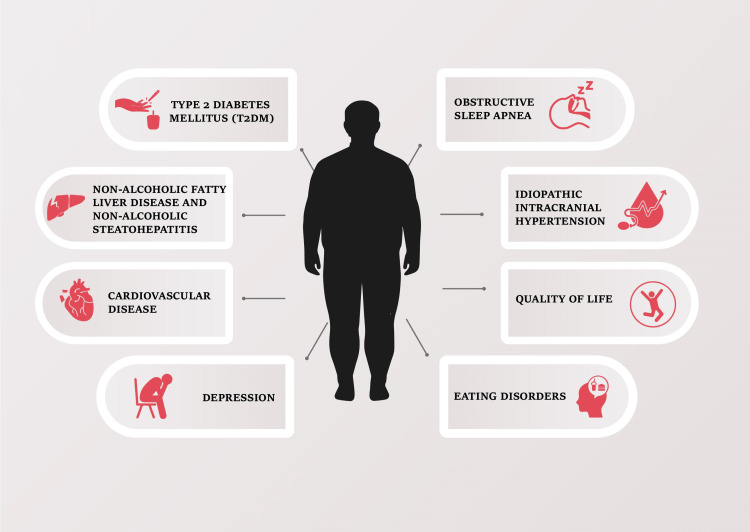
The Comorbidities Associated with Obesity Among Children and Adolescents. Adapted from the American Society for Metabolic and Bariatric Surgery (ASMBS) Public Education Committee and the ASMBS Pediatric Committee [10].

Recent guidelines from the ASMBS state that Tanner staging and linear growth are not factors to consider when evaluating teenagers aged 10-19 years with Spartan obesity (session 2 with comorbid condition or session 3) for MBS [3]. These guidelines also provide an assessment of the common risks of obesity, as seen in Figure 2. Despite the strong evidence supporting the effectiveness and safety of MBS, concerns regarding long-term and peri-operative consequences, lack of experience in management, and the potential risk of mineral and vitamin malabsorption, which can lead to reduced growth, have limited MBS as a viable option for children and adolescents [15,16]. These barriers mean that children are often not referred to MBS programs until their obesity has progressed significantly. Comorbidities associated with childhood obesity can have severe and potentially irreversible impacts on children's development if left untreated [17].

Various methods, including dietary modifications, increased physical activity, behavioral therapy, and medicines, are employed in managing obesity in individuals of all ages. Adolescents are often prescribed lifestyle changes followed by medication for weight management, as there are limited treatments authorized for this population [18]. However, these methods often fall short of achieving future weight maintenance, which is crucial for improved health outcomes [19,20]. Surgeries such as the sleeve gastrectomy (SG) and gastric bypass (RYGB) have shown promising results in managing severe obesity in adolescents, with an overall weight loss of 23-28% at one year and 16-22% at five years compared to nonsurgical patients [21].

The RYGB procedure has been a longstanding and commonly used method for treating obesity in adolescents. First developed in 1967, laparoscopic RYGB has been performed since 1993. Prior to 2014, it was considered the gold standard for pediatric surgery. Adolescents who undergo RYGB have shown significant improvements in health. The Adolescent Morbid Obesity Surgery (AMOS) study included 81 adolescents who met the criteria for MBS. Results showed that up to five years after surgery, 90% of patients reported a BMI reduction of 13.1 kg/m^2^ (or a 29.0% decrease in total body weight). However, 25% of patients required additional surgery due to weight regain [22].

An analysis of data from 2005 to 2015, conducted by Arafat and colleagues [23], found that out of the 2,625 patients aged 18-21 years who underwent MBS, 43.4% received RYGB while 32.5% underwent SG. In the 2018 edition of the recommendations, the ASMBS suggests that SG is the preferred method for treating adolescent obesity [3].

A study by Dewberry et al. [24] determined that after a five-year monitoring period, patients who underwent SG were four times more likely to experience gastroesophageal symptoms, in comparison to those who had undergone RYGB (161 vs. 67). As such, it is important for patients to be informed of the potential for reflux problems before undergoing SG, and for doctors to closely monitor them after surgery.

Teen-LABS was a large study that aimed to investigate the results of bariatric surgery performed on adolescents with obesity. The study revealed a significantly reduced BMI at a three-year follow-up in the intervention group, highlighting the effectiveness and success of bariatric surgery for obesity management [25,26]. The decrease in BMI was 28% (95% CI, 25-30) in individuals who underwent RYGB and 26% (95% CI, 22-30) in those who had SG. The findings demonstrate that bariatric surgery could result in sustained weight loss, which is a significant accomplishment for patients with obesity.

The SG is the preferred method for treating severe obesity in adolescents, surpassing RYGB. The decision to recommend a specific surgery should consider patient factors, including the potential benefits and risks of neurocognitive therapy (RYGB) versus gastric restriction surgeries (gastric sleeve, banding) [27]. Bariatric surgeries lead to weight loss in addition to the resolution of comorbidities. Studies show that patients who underwent bariatric surgery experienced significant reductions in health issues related to obesity like hypertension, dyslipidemia, and proteinuria, as compared to those who received medication and lifestyle adjustments [28]. The Teen-LABS study reported a significantly reduced BMI at the three-year follow-up in adolescents treated with bariatric surgery, with RYGB resulting in a greater decrease (28%) than SG (26%) [25,26]. Furthermore, surgical procedures have been found to alleviate obesity-related comorbidities in various trials [29,30].

Recent research has explored weight stigma in youth, revealing that childhood obesity is associated with negative effects such as peer victimization, poor health, academic difficulties, and social isolation [31]. A study conducted in 20 schools found that 27% of young individuals reported being bullied due to their weight [32]. Furthermore, research has shown that weight-related stigma affects not only overweight children but also those who are underweight. A survey revealed that 64% of underweight individuals had experienced weight-related stigma, with 71% reporting facing such stigma in school during the previous academic year [33].

Given that MBS can result in substantial health improvements, the bariatric community has recently shown an interest in SG due to its lower technical demands and reduced malabsorption of certain nutrients compared to RYGB. While research in this area has yielded mixed results [34,35], early findings with adolescent SG are promising and consistent with those seen in adult research. As a result, SG is the preferred technique for bariatric centers providing surgery to patients in this age group [36].

Barriers in MBS

Regional variations in the availability of pediatric obesity experts pose a significant barrier to the delivery of appropriate and adequate treatment for children with obesity. The scarcity of practitioners trained in the surgical treatment of childhood obesity is a persistent issue, with fewer American Board of Obesity Medicine (ABOM) diplomats than those competent to treat adult obesity, as described by Gudzune et al. [37]. This lack of trained pediatricians is particularly challenging in densely populated areas.

Additionally, the shortage of bariatric surgeons experienced in treating younger adolescents and pediatricians with advanced training is another significant barrier. Several organizations, such as the American College of Surgeons, the Society for Surgery of the Alimentary Tract, and the Society of American Gastrointestinal and Endoscopic Surgeons, have collaborated to establish standards for credentialing bariatric surgeons. These organizations recommend that surgeons train at established programs and complete a fellowship in bariatric surgery at an accredited institution. Only a limited number of surgeons specialize in bariatric surgery, and most only treat adults, resulting in low numbers of individuals undergoing the procedure [38].

Telehealth as a useful tool in addressing pediatric obesity

Telehealth is becoming very important for the management of pediatric obesity, as it allows obesity prevention programs to reach individuals regardless of their location or logistical challenges. Telehealth may be particularly beneficial for remote care by providing clinicians with baseline information such as a patient's eating and exercise habits. Additionally, the use of telehealth for post-bariatric surgery mental follow-up and symptom therapy is promising and has the potential to improve outcomes, regardless of a patient's socioeconomic status [39].

A recent study also showed that telehealth is safe and effective for postoperative care, further indicating its potential as an ideal tool for pediatric obesity management [40]. Formal surveys of 123 patients who utilized virtual clinics during the COVID-19 pandemic revealed a high level of satisfaction, particularly within the dermatology and psychiatry clinic setting [41]. This technology can provide a realistic, low-cost, and easily accessible option for pediatric patients to engage with a weight management team, improving access to care [42].

However, despite all the benefits provided, there are some limitations that must be considered. To ensure the success of telehealth, operational facilities must have sufficient technology, and adequately trained staff which can incur additional costs. Although telehealth has the potential to enhance the management of obesity by removing logistical barriers for children who are overweight to receive care, telehealth alone cannot solve the fundamental problem of a lack of bariatric surgeons in rural locations. It is important to recognize that telehealth cannot replace in-person care completely and that there are still challenges to overcome, such as the shortage of healthcare professionals with specialized training in pediatric obesity management.

## Conclusions

Childhood obesity continues to remain a significant public health issue linked to detrimental health complications. Treating childhood and adolescent obesity is particularly important to prevent the likelihood of health problems as an adult. While lifestyle changes are crucial, weight loss surgery, or MBS, is a viable solution for some children and adolescent patients living with severe obesity. Based on the findings of this literature review, MBS can provide significant benefits. The surgery has been associated with substantial weight loss amongst improvements in comorbidities and overall quality of life. These guidelines provide an essential framework for management. Moreover, telehealth is identified as an increasingly valuable tool for managing pediatric obesity, as it can improve access to care, particularly for those in remote areas. However, it is acknowledged that telehealth cannot replace in-person care completely, and there are still challenges to overcome. Healthcare practitioners should take note of these guidelines and recognize the importance of patient management by utilizing a multidisciplinary team approach. To help achieve the best results for the patients, it is also critical for different types of experts to work together as a team and to have a plan for referring patients to specialized facilities focused on treating obesity in the pediatric population.
